# Methods of a multi-faceted rapid knowledge synthesis project to inform the implementation of a new health service model: Collaborative Emergency Centres

**DOI:** 10.1186/2046-4053-4-7

**Published:** 2015-01-14

**Authors:** Jill A Hayden, Lara Killian, Austin Zygmunt, Jessica Babineau, Ruth Martin-Misener, Jan L Jensen, Alix J Carter

**Affiliations:** Department of Community Health and Epidemiology, Dalhousie University, 5790 University Avenue, Halifax, NS B3H 1V7 Canada; Nova Scotia Cochrane Resource Centre, Dalhousie University, 5790 University Avenue, Halifax, NS B3H 1V7 Canada; Toronto Rehabilitation Institute, University Health Network, 550 University Avenue, Toronto, ON M5G 2A2 Canada; School of Nursing, Dalhousie University, 5869 University Avenue, Halifax, NS B3H 3J5 Canada; Emergency Health Services, 239 Brownlow Avenue, Suite 300, Dartmouth, NS B3B2B2 Canada; Department of Emergency Medicine, Division of Emergency Medical Services, Dalhousie University, QEII Health Sciences Centre, Halifax Infirmary, Room 3019, 1796 Summer Street, Halifax, NS B3H 3A7 Canada

**Keywords:** Rapid review, Jurisdictional review, Integrated knowledge translation, Evidence synthesis, Collaborative Emergency Centres, Rural health care

## Abstract

**Background:**

The aim of this rapid knowledge synthesis was to provide relevant research evidence to inform the implementation of a new health service in Nova Scotia, Canada: Collaborative Emergency Centres (CECs). CECs propose to deliver both primary and urgent care to rural populations where traditional delivery is a challenge. This paper reports on the methods used in a rapid knowledge synthesis project to provide timely evidence to policy makers about this novel healthcare delivery model.

**Methods:**

We used a variety of methods, including a jurisdictional/scoping review, modified systematic review methodologies, and integrated knowledge translation. We scanned publicly available information about similar centres across our country to identify important components of CECs and CEC-type models to operationalize the definition of a CEC. We conducted literature searches in PubMed, CINAHL, and EMBASE, and in the grey literature, to identify evidence on the key structures and processes and effectiveness of CEC-type models of care delivery. Our searches were limited to published systematic reviews. The research team facilitated two integrated knowledge translation workshops during the project to engage stakeholders, to refine the research goals and objectives, and to share interim and final results. Citations and included articles were categorized by whether they addressed the CEC model or component structures and processes. Data and key messages were extracted from these reviews to inform implementation.

**Results:**

CEC-type models have limited peer-reviewed evidence available; no peer-reviewed studies on CECs as a standalone healthcare model were found. As a result, our evidence search and synthesis was revised to focus on core CEC-type structures and processes, prioritized through consensus methods with the stakeholder group, and resulted in provision of a meaningful evidence synthesis to help inform the development and implementation of CECs in Nova Scotia.

**Conclusions:**

A variety of methods and partnership with decision-makers and stakeholders enabled the project to address the limitations in the evidence regarding CECs and meet the challenge of identifying the best available evidence in a transparent way to meet the needs of decision-makers in a short timeframe.

## Background

Health policy decision-makers are often tasked with designing and delivering complex programs and services that meet the needs of a population. Research evidence is important to inform and guide appropriate decision-making [1]. Evidence-informed decision-making uses transparent and systematic processes in which policy decision-makers use research evidence to help clarify a problem and to identify and implement options to address it [2–4]. The effective integration of research evidence can lead to more successful programs and practices and can subsequently improve health outcomes [5].

However, overcoming challenges in using research evidence in policy decision-making, including finding evidence that addresses the question of interest, unclear perceived relevance to the local context, and compressed timelines for decision-making, requires focused and innovative knowledge syntheses and knowledge translation approaches [6]. Policy decision-makers need relevant summaries of research evidence to help address complex health and health system problems [7]. Rapid reviews are an emerging approach to produce rigorous yet pragmatic summaries that respond to the challenge of restricted timelines [8]. Compared to systematic reviews, such as Cochrane Reviews, the methods of rapid reviews follow a more streamlined approach to produce an evidence summary over a shorter timeframe (2–6 months rather than 12 months or more, which is typical for a systematic review [8, 9]).

While systematic reviews and rapid reviews can provide useful summaries of ‘What works?’ , the integration of evidence into decision-making remains limited [7]. Regardless of the preferred type of knowledge or quality of evidence available for decision-making purposes, consistency in the literature is an acknowledgement that closer partnerships between researchers and decision-makers are crucial [10, 11]. Integrated knowledge translation (KT) involves the reciprocal exchange of information and enhanced collaboration between researchers and decision-makers and has been identified as a factor that influences the positive use of available research evidence. These iterative researcher/decision-maker partnerships involve the users of research in the conduct of the research, facilitating an exchange of information between researchers and decision-makers, providing gains in practical and conceptual knowledge for both, and enhancing the mutual understanding and relevance of the research and decision-making processes [7, 12–18].

Despite the increased production of rapid reviews and projects with an integrated decision-maker involvement, there is minimal methodological guidance available for these approaches [19]. In addition, few projects transparently report their methods to allow replication. Two recent studies have assessed examples of rapid review methodologies and found no consistent use of methods [8, 20]. In order to develop a standardized methodology for rapid reviews, Ganann and colleagues (2010) recommended that future rapid reviews not only provide condensed reports for decision-makers but also publish a separate paper with an emphasis on the methodology used [8]. In this paper, we present an example of a project that included a jurisdictional/scoping review, rapid review, and integrated KT component summarizing evidence for a new complex healthcare delivery model in our region: the Collaborative Emergency Centre (CEC). We describe the researcher/decision-maker partnership, review methods, outcomes, and evaluation.

### Project example: Collaborative Emergency Centres—a rapid knowledge synthesis project

CECs were introduced in the province of Nova Scotia, Canada in 2011 in response to the challenges of access to emergency and primary care services in some rural areas of the province. The goal was to ‘keep emergency departments open, reduce patient wait times, and provide a team-based approach that offers continuity of care’ [21–25]. CECs are an innovative healthcare delivery model that brings nurses, doctors, paramedics, and other healthcare providers together in one location to provide access to timely urgent and primary care. Although the Nova Scotia Department of Health and Wellness had committed to opening Nova Scotia’s first CEC in April 2011, prior to commissioning the synthesis project, they wished to inform the development and implementation of effective CECs by understanding the available evidence on this unique model and options to consider in implementation. The first CEC opened in July 2011, with four additional CECs announced in October and November of the same year.

Our research team was engaged to:

1. Define CECs through the identification of potential structures, processes, and implementation strategies of CEC-type models in other jurisdictions.
2. Identify scientific evidence that investigates the effectiveness of CEC-type models and their structures and processes for improving health outcomes.

Our team worked with the government policy advisors and the Nova Scotia Health Research Foundation (http://www.nshrf.ca) to inform this complex healthcare and service delivery problem. Challenges to informing this topic included starting with a broad question of interest and limited research evidence on the full model of care. Regardless, there was a need to inform practice. In response to these challenges, a variety of methods were adopted for the project, which permitted the synthesis of the evidence on the core components of collaborative health models and collaboration with decision-makers to refine aspects of the project focus. This approach permitted opportunities for conversations about project methods, research goals, and availability and use of research evidence.

## Methods

### Overview of project methods

**Figure 1:**
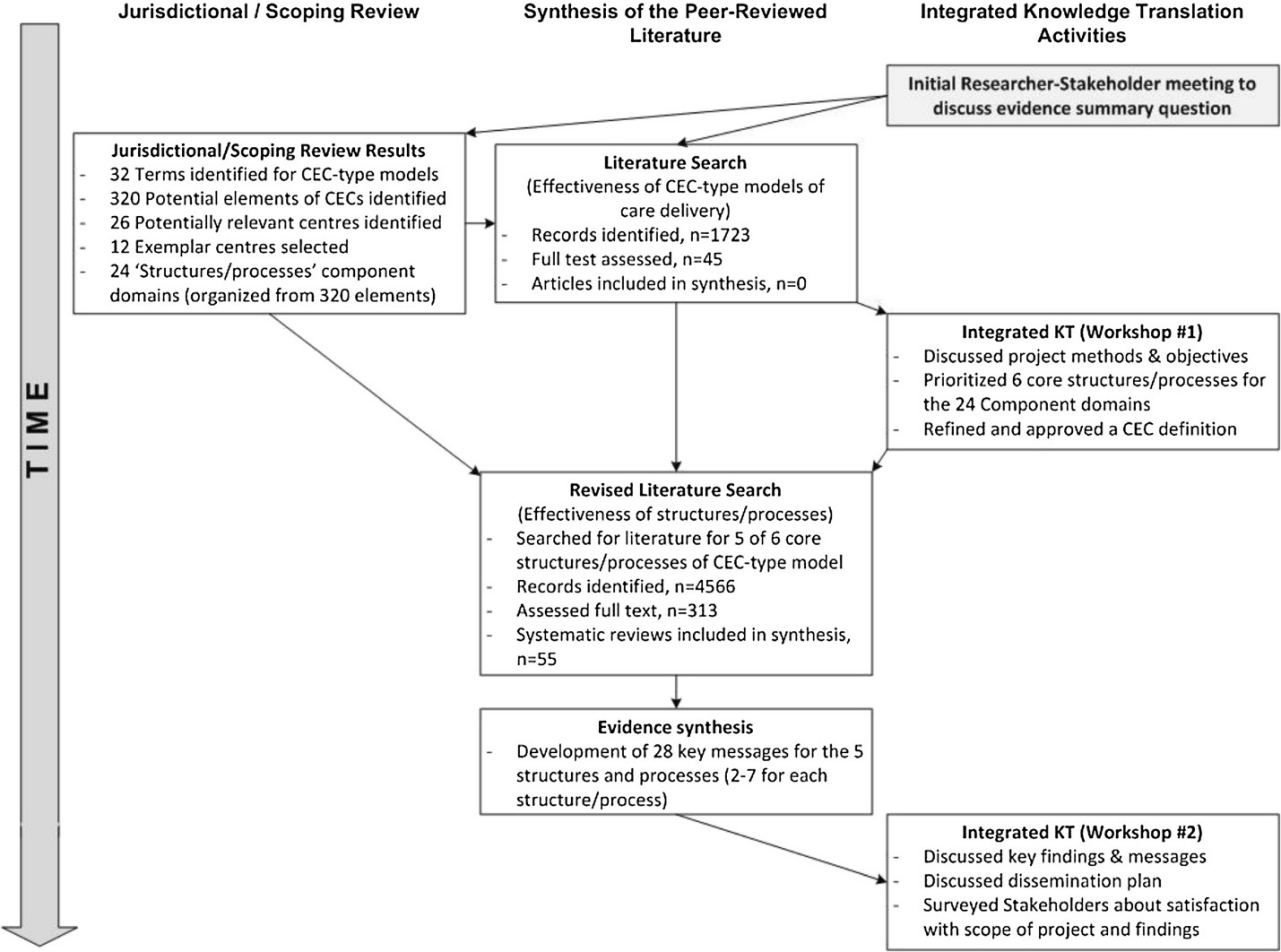
**Flow chart of rapid knowledge synthesis project components.**

In choosing the methods for this project, we considered the required timeframe of a synthesis, the complexity of the topic area and the necessity of producing a summary of value relevant to decision-makers in Nova Scotia. We adapted methods for rapid reviews (emphasizing expedited systematic review methods) [8, 19], including a jurisdictional/scoping review and used an integrated KT approach [26]. The project was conducted over a 6-month timeframe. Figure 1 describes the overall flow of the project components.

The proposal for this project was reviewed by the Capital Health Research Ethics Board and was determined not to require ethical approval and informed consent.

### Developing the team

The rapid knowledge synthesis project was led by the members of the Nova Scotia Cochrane Resource Center. A team of methodology and content experts was recruited. Our team’s expertise included epidemiology, health sciences, literature searching, evidence synthesis, and project management. A nurse practitioner, an emergency physician, and an advanced care paramedic joined the team as content experts. A research assistant trained as an emergency department nurse assisted with scoping the literature about the model of care.

### Jurisdictional/scoping review

We conducted a review of relevant practices and models in other jurisdictions in Canada to help map out the complex concept of a CEC [27]. The objectives of the jurisdictional/scoping review were to investigate the following:

1. What CEC-type models have been applied in jurisdictions outside of Nova Scotia?
2. What components are included in these healthcare delivery models?
3. How are CEC-type models assessed (e.g. what outcome measures are used)?

The information from the jurisdictional/scoping review was used primarily to inform and operationalize the definition of CEC-type models to guide the scientific evidence search and synthesis rather than to inform the implementation of CECs in Nova Scotia. Initial scoping searches led to a list of 32 terms that describe healthcare delivery models similar to CECs, combining primary and emergency care in a single setting; these healthcare delivery models included, for example, ‘multidisciplinary centre’, ‘polyclinic’, ‘satellite clinic’, and ‘wellness center’. We collected information on these models by scanning publically available information online and confirming information by telephone, with site representatives. Canadian jurisdictions were selected for further exploration if they had a similar model of care and similar demographics to our Nova Scotia context.

Digital resources were selected for relevant individual jurisdictions by a librarian researcher. Resources investigated included government and health department websites, individual health authority websites, research citation databases, and Google search engines (Google Scholar, Google News). We selected relevant centres based on our CEC-type model definition, which was refined through a consultation with investigators and stakeholders. Potential centre descriptions were assessed by two researchers with conflicts resolved through a consultation among the research team. We contacted centres with particular relevance to the Nova Scotia context (i.e. had a similar model of urgent and primary care in one setting and similar population demographic characteristics). We clarified our understanding of individual jurisdictional practices, as described through web presences, with centre representatives by telephone. Information from telephone contact was integrated into the data extraction. We compiled a comprehensive set of 320 distinct elements potentially relevant to CECs, which were grouped into 24 searchable component domains. The subsection, ‘Important topics relevant to CECs’ lists the 24 components searched. This scoping activity defined important considerations for CEC-type models and directed our topics for searching the evidence and evidence synthesis. During this process, we iteratively refined an operational definition of a CEC, which was unavailable prior to this exercise. The final operationalization of a CEC is outlined in the subsection, ‘Definition of a Collaborative Emergency Centre’.

### Important topics relevant to CECs

Here is a list of topics identified as important for informing implementation of CECs, including the evidence of effectiveness of CEC-type models, and relevant structures and processes.

Original topic of interest:

- Full CEC-type models*

Prioritized component topics:*

- Hours of access to emergency or primary care services
- Health care professional staff available in an emergency care
- Health care professional staff available in a primary health care
- Collaborative practices in emergency or primary health care delivery
- Tele-health or teleconsultation
- Diagnostic services available in emergency or primary health care*

Non-Prioritized component topics*Revised literature searches and evidence synthesis occurred for five of the six prioritized components. The full CEC model remained a priority item despite a lack of evidence. Lack of a manageable, comprehensive search strategy for diagnostic services meant that this component could not be efficiently searched or synthesized within this rapid knowledge synthesis.

- Structure of emergency services (i.e. triage)
- Structure of primary care services (i.e. walk-in, same day availability)
- Emergency protocols or use of standing orders
- Destination and transfer plan
- Service infrastructure
- Ambulatory clinic services
- In-patient beds available
- Formal community health needs assessment
- Health promotion & prevention services
- Specific governance structure
- Formal program evaluation
- Specific program funding structure
- Specific funding structure for health professionals
- Community awareness campaigns
- Recruitment and retention programs
- Affiliation with an educational institution
- Involvement in conducting research

### Definition of a Collaborative Emergency Centre

As operationalized by clinical stakeholders and policy decision-maker partners:

- A CEC-type centre focuses on the delivery of health care services including access to both primary care and emergency care through a seamless collaborative team approach.
- Primary care encompasses access to health promotion, wellness, chronic disease management, illness and injury prevention, and diagnosis and treatment of illness and injury.
- Access to emergency care includes initial emergency stabilization of life-threatening conditions, response to (including treatment or referral) the majority of urgent conditions and those conditions of lesser urgency.

A health care provider must be available on-site, and has a formal supportive relationship with other professional(s) or institution(s) elsewhere through telephone or technological means.

### Integrated knowledge translation

We used an integrated KT approach, engaging clinical and policy decision-makers, to help refine our evidence summary questions and interpret findings relevant for CECs in Nova Scotia. This was executed through two half-day workshops with selected local stakeholders, including individuals from groups relevant to CECs in Nova Scotia and able to provide policy, operations, administration, and healthcare provider perspectives. This included 19 representatives from the Department of Health and Wellness, primary healthcare practitioners (family physicians, nurses), emergency care practitioners (members of Emergency Health Services, emergency physicians), and members of the Cumberland County Health Authority involved in the development and/or functioning of the first implemented CEC [28]. Workshops were designed with activities and small group discussions to engage attendees, to encourage discussion, and to reach consensus. We encouraged free expression of questions, concerns, or comments about the methods or results within and outside of the workshops.

#### Integrated knowledge translation—research phase

A first stakeholder workshop was held once the project was underway in which CEC-model components identified in the jurisdictional/scoping review were presented. The goals of the stakeholder workshop were to discuss methods for evidence synthesis in general; to discuss the objectives and approach of our specific project; to refine definitions and priorities within the project; and to discuss key findings, key messages, and dissemination plans. The revised goals developed within the first workshop were (1) to define CECs through the identification of potential structures, processes, and implementation strategies of CEC-type models; and (2) to identify scientific evidence investigating the effectiveness of CEC-type models and their structures and processes.

The set of 24 structures and processes, identified in the jurisdictional/scoping review, were presented at the first workshop. Stakeholders identified the components that they felt were most important for the model being implemented in the province. Prioritized components were identified through a consensus exercise: sheets of paper with individual structures and components were placed on the wall of the meeting room; everyone receiving an equal number of small dot stickers; attendees placed their dot stickers next to the structure or processes they considered most important; results were tallied and six structures and processes were clearly identified as the most important to consider, in addition to the overall concept of a CEC. These were prioritized as having critical importance to the functioning and local implementation of a CEC. A subsequent evidence synthesis was focused on these components (subsection, ‘Important topics relevant to CECs’).

### Rapid review

#### Conducting the review of the published literature

Comprehensive literature searches in peer-reviewed and grey literature were executed for each prioritized component (structures/processes). Given the broad nature of this review project and the limited time available to summarize the findings from all prioritized research questions, the primary goal was to identify recent, high-quality systematic reviews relevant to CECs and CEC-type models and each prioritized structure or process. Therefore, search strategies were first conducted with an established filter, if available (e.g. PubMed filter [29] to identify systematic reviews). All searches were limited to research published in the English language. We did not restrict the search by the year of publication.

If no relevant systematic reviews were identified, the search was repeated without the systematic review filter to identify relevant primary studies. If no primary studies were identified, a grey literature search (guided by the GreyMatters tool from the Canadian Agency for Drug and Technologies (CADTH) [30]) was conducted between 18 and 21 October 2011.

The objectives of the rapid evidence synthesis were to describe the following:

1. The effectiveness of CEC-type models compared to traditional healthcare delivery models, and
2. The effect of specific CEC-type model structures or processes.

We imported citations identified in the searches into Endnote X4 reference management software [31], removed duplicate citations, and uploaded citations into DistillerSR web-based systematic review management software [32]. Screening was conducted in three stages. In stage 1, a single reviewer screened the titles, discarding any that were clearly irrelevant. All other articles advanced to the next stage of citation screening. In stage 2, two independent reviewers screened the abstracts using an accelerated screening approach: articles advanced to full text consideration (stage 3) if one of the two reviewers judged the abstract to be relevant, while the consensus of two reviewers was required to reject an abstract. In stage 3, we retrieved and screened full text articles available electronically through Dalhousie University Libraries, which has substantial holdings in the medical literature due to the presence of a Faculty of Medicine at the University (the project timeframe did not include time to retrieve articles by interlibrary loan). Studies flagged as not relevant in full article review were checked by a second reviewer before exclusion.

1. *Effectiveness of CECs:* For the overall question about the effectiveness of CEC-type models of healthcare delivery on health outcomes, we identified and screened 1,723 unique citations as title and abstract records and excluded 1,678 records. At the full text level, we screened 45 results available electronically; none of these met our criteria for inclusion in our summary of evidence. Zero reviews or studies were identified that assessed the effectiveness of CEC-type models as a complete concept. We identified 12 grey literature sources potentially providing evidence on the effect of CEC-type models of care delivery. Following thorough assessment, these 12 grey literature documents were also excluded.
2. *Effect of Structures/processes:* To overcome the obstacle of a lack of evidence for ‘CECs’ from our initial database and grey literature search, we searched for evidence on the processes and structures identified in the jurisdictional/scoping review and prioritized by the researchers, content experts, decision-makers, and stakeholders in the first integrated KT workshop. The new search strategy included terms to capture the prioritized component parts and structures and processes for CEC-type models with the goal to gather evidence about the feasibility and potential effect of the component parts of such model.

An information specialist developed and executed comprehensive literature searches for systematic reviews on five of the six prioritized CEC structures and processes. PubMed, EMBASE, CINAHL, and The Cochrane Library (including Cochrane Reviews and other reviews indexed in DARE, the Database of Abstracts of Reviews of Effects) were searched between 15 August and 20 September 2011 for each prioritized structure or process question. Full search strategies for database searches for prioritized structures and processes are available on request. No date limits were set. One prioritized structure, ‘having a comprehensive set of diagnostic services available in primary or emergency care’, was not included in the knowledge synthesis as we were not able to develop a manageable, comprehensive search strategy for this concept.

**Figure 2:**
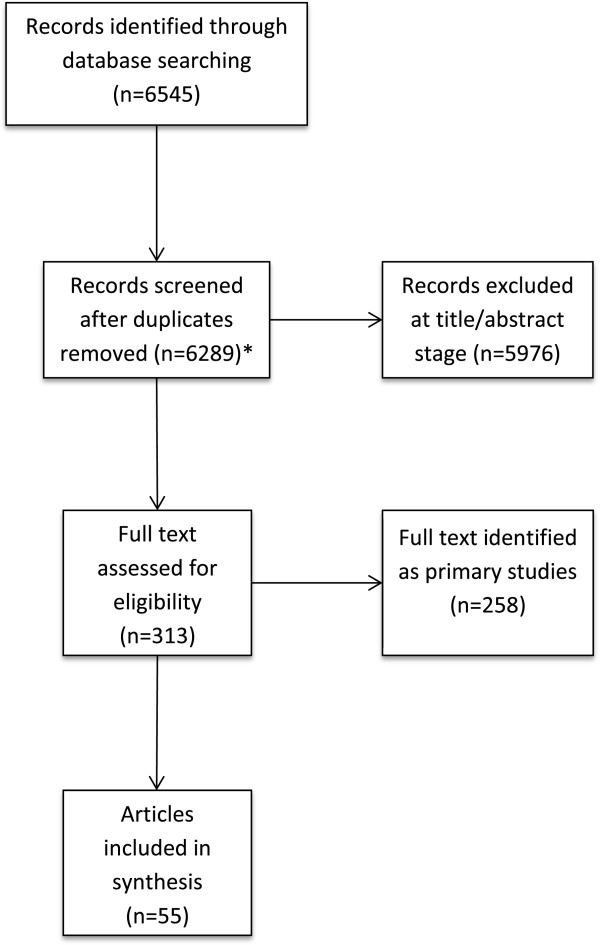
**Flow chart of database search and study screening results for all prioritized questions.** *Includes 1723 unique articles identified for CEC-type models and 4566 unique articles identified for the five prioritized structures and processes searched.

Four thousand five hundred sixty-six unique citations were identified and screened as title and abstract records. Four thousand two hundred fifty-three records were excluded in stages 1 and 2. In stage 3, we retrieved and screed 313 full text articles, excluding 258. Fifty-five systematic reviews were included in our summary of evidence. A range of 2–19 systematic reviews were identified for each of the prioritized CEC structures/processes (see Figure 2 for a flowchart of database search and screening results). One researcher extracted data from full text articles into the DistillerSR software. One additional researcher reviewed the data extraction for accuracy. Data items extracted from the included studies are listed in the subsection, ‘Data items extracted from the included studies’. We exported the collected data to Microsoft Excel to format and summarize into tables for the team to review.

Data items extracted from the included studies:

- Author objectives
- Description of participants/setting, interventions, and outcomes (related to health, process, and/or costs)
- Author conclusions
- Review quality (type of evidence, potential bias, generalizability)
- Quality of evidence included

#### Synthesis of the evidence

There was insufficient homogeneous data to allow quantitative synthesis of results. Instead, the content experts developed key messages and synthesized overall messages from the data extracted from the systematic reviews and studies included in the synthesis for the prioritized CEC structures and processes. They used worksheets developed to extract key messages on the effectiveness of each prioritized CEC structure or process. Content experts flagged any records that they felt were not fully relevant to the specific structure or process, or which they believed were not generalizable to our local Nova Scotian context; these studies were discussed by the review team and excluded by a consensus decision. A minimum of two content experts looked at each data table and extracted the messages. The review team compared the results and assessed them for consistency. Twenty-eight key messages were identified from the evidence assessed in our review. This included a range of 2–7 (median =5) key messages extracted for each of the prioritized searches. Examples of key messages are provided in subsection, ‘Examples of key messages’.

### Examples of key messages

Selected examples of key messages extracted from the included systematic reviews and studies identified through a comprehensive search of the published literature.

- There is good evidence from systematic reviews to support Nurse Practitioners working in primary care and in the ED with regard to patient satisfaction and compliance, with care at least equivalent to a physician-in-training.
- This review captured limited evidence from systematic reviews on the role of paramedics in the ED or primary care.
- There is limited but promising evidence from systematic reviews on the benefit of tele-consultation.
- Cost-effectiveness of these novel approaches is unknown.
- A growing number of CEC-type models are in operation across Canada and the world; Nova Scotia has the opportunity to share our experiences and to lead.

### Integrated knowledge translation—dissemination phase

Key messages and the data tables were discussed at a second stakeholder workshop. A checklist of structures and processes of CECs was provided to stakeholders to inform future implementation of new CECs.

Feedback was collected from stakeholders on their satisfaction with the project scope and findings in the form of a brief anonymous survey collected on paper at the end of the workshop. Stakeholders were asked to comment on the project following the final presentation. Comments included ‘Excellent work compiling available research’, ‘Very informative for decision-making’, ‘It was collaborative with stakeholders’, and also ‘We need to better define our questions so we can have the research assist us to make operational level decisions’. When asked, ‘In your opinion, how useful will information provided today be for decision-making with regard to Collaborative Emergency Centres?’, 100% of participants responded ‘somewhat useful’ or ‘very useful’.

Three reports were produced an executive summary (2 pages), a short report (16 pages) [33], and a full report (107 pages) [34]. The reports are available on the Nova Scotia Health Research Foundation website (http://www.nshrf.ca/sites/default/files/cec_rapid_knowledge_synthesis_short_report_2012.pdf) and (http://www.nshrf.ca/sites/default/files/cec_rapid_knowledge_synthesis_full_report_2012.pdf) (accession date 14 November 2012).

## Results and discussion

We encountered several challenges in this project that are not atypical of initiatives to apply research evidence to policy decision-making. These included a broad proposed question on a complex healthcare delivery topic, and no direct research evidence was available. The establishment of CECs in Nova Scotia sought to fulfill a key commitment of the *Better Care, Sooner* report by helping to address issues of emergency department overcrowding and long wait times to see general practitioners [35] and to facilitate a Department of Health and Wellness objective to ‘keep EDs open, reduce patient wait times, and provide a team-based approach that offers continuity of care’ [36–40]. The limits for guiding implementation of CECs were initially only to satisfy this objective. Furthermore, as a novel health delivery strategy, the term ‘Collaborative Emergency Centre’ did not exist in the peer-reviewed or grey literature. It was therefore necessary to think critically about what core components define a CEC and how these elements might be described in the literature.

Our approach to meet these challenges was to clarify the operationalization of the healthcare delivery model, to break the model into component parts to search for evidence, and to prioritize structures and processes with stakeholders for the evidence summary. This approach could be useful to inform development and implementation of other unique, complex, or multicomponent healthcare services. Initial scoping searches led to a list of names and terms that describe healthcare delivery models similar to CECs. We worked with local content experts to identify the most useful evidence for our context. This helped to provide the best evidence available for the policy issue at hand. Consensus of key clinical stakeholders and health policy decision-makers on a definition of CECs to clarify the scope for communication was an important outcome for the project. The subsequent evidence synthesis was based on research evidence obtained from the revised search.

Due to the complexity of the topic of interest and time constraints, our research team applied a rapid review approach with goals of transparency and practical relevance in mind. The rapid knowledge synthesis project used systematic and transparent methods that minimize potential bias. We streamlined the search and selection procedures by using an accelerated process, and focused on systematic review evidence when available, and studies published in the English language. We used similar methods to the ‘rapid review’ approach described by Khangura et al. [19], although longer in duration. We aimed to complete this project in four months; however, our project took six months to complete. Although abbreviated methods of a rapid review may lead to a potential bias in results (e.g. publication bias related to selection of English language documents and omission of primary research articles), selecting systematic reviews allowed us to focus on high-quality evidence that was already synthesized. A limitation of this approach is that evidence from primary studies that were relevant, but not included in published systematic reviews, was not included in our synthesis, which may be an important consideration when seeking evidence on such novel services like CECs.

In addition to summarizing the available evidence, this project developed researcher/policy decision-maker relationships and provided a local example of the potential value of using research evidence to inform decision-making. Including integrated KT components added to our timeline, however, we think that this was an important part of our project. Two factors that substantially increase the likelihood of health policy related research being used to inform policy are engagement between researchers and policy makers and timeliness of delivery of information [10]. We used integrated KT with key decision-makers and stakeholders to clarify and inform the complex question of interest and summarized research evidence to inform decision-making in the context of lack of evidence on the specific healthcare delivery approach. The workshops held throughout the project provided opportunities to interact with and to educate policy decision-makers and other stakeholders about the research evidence. Our partnership between researchers and policy decision-makers enhanced the project and facilitated revising the project goals and search strategies to provide a meaningful evidence synthesis to help inform the development and implementation of CECs in Nova Scotia. Furthermore, relationships between researchers and policy decision-makers have facilitated subsequent collaborations, which we think will increase evidence-informed decision-making in our region. The inclusion of stakeholders from similar centres in other jurisdictions and users of the health services in the CEC regions would have further enriched the project.

An important limitation of our project was that we did not comprehensively evaluate practical application of our evidence synthesis approach and did not assess the impact of the stakeholder interactions and evidence report. Future researchers should consider a parallel investigation that evaluates the usefulness and impact of policy-related evidence synthesis projects and decision-maker interactions on decision-making and the implementation of health services.

The information and framework provided by our knowledge synthesis informed subsequent research projects evaluating CECs in our region. Ongoing research is assessing the impact of CECs and effect of specific healthcare delivery components, guided by our review. Policy decisions in our region, and other Canadian jurisdictions, are waiting for the results of these evaluations.

## Conclusions

Through participating in this first rapid knowledge synthesis, our research centre further developed existing and new relationships with provincial health policy decision-makers and clinicians. By working directly with these stakeholders, we were able to address limitations in the available evidence and identify areas where further knowledge was prioritized. This is one example of a rapid review project with a goal of providing useful and timely research evidence for decision-makers. We based our approach on available guidance and attempted to follow principles of transparency, limiting bias, and integrated KT. However, there remains a need for future studies to evaluate rapid review methods and to assess the effectiveness of different approaches to increase decision-makers’ use of research evidence.
